# Racial disparities in hepatocellular carcinoma: a TCGA-based gene expression study of Caucasian and Asian populations

**DOI:** 10.37349/etat.2025.1002344

**Published:** 2025-11-02

**Authors:** Muhammad Rezki Rasyak, Sri Jayanti, Cyrollah Disoma, Bens Pardamean, Caecilia Sukowati

**Affiliations:** Chinese Peoples Liberat Army China (PLA) General Hospital, China; ^1^Bioinformatics and Data Science Research Center, Bina Nusantara University, Jakarta 11480, Indonesia; ^2^Eijkman Research Center for Molecular Biology, National Research and Innovation Agency (BRIN), Central Jakarta 10340, Indonesia; ^3^Liver Cancer Unit, Fondazione Italiana Fegato ONLUS, AREA Science Park, Campus Basovizza, 34149 Trieste, Italy; ^4^Doctoral School of Molecular Biomedicine, Department of Life Sciences, University of Trieste, 34149 Trieste, Italy; ^5^Computer Science Department, BINUS Graduate Program - Master of Computer Science, Bina Nusantara University, Jakarta 11480, Indonesia

**Keywords:** hepatocellular carcinoma, gene expression, populations, TCGA

## Abstract

**Aim:**

Hepatocellular carcinoma (HCC) displays both shared and ethnicity-specific molecular characteristics. Using transcriptomic data from The Cancer Genome Atlas (TCGA), we compared gene expression profiles between Asian and Caucasian HCC patients.

**Methods:**

Gene expression profiles were analyzed using the PyDESeq2 implementation of DESeq2, applying size factor normalization and dispersion estimation. Differentially expressed genes (DEGs) were identified with thresholds of false discovery rate (FDR) of < 0.05 and |log_2_FC| ≥ 1.0. Gene annotation, visualization, and pathway enrichment were conducted using Sanbomics, seaborn, and gene set enrichment analysis (GSEA) via the GSEApy package.

**Results:**

A total of 387 and 250 genes were commonly upregulated and downregulated, respectively, in both populations, including the upregulations of *GPC3* and *PLVAP* and the downregulations of *FCN3* and *OIT3*, indicating their potential as universal HCC markers. Conversely, 16 genes were upregulated in Asians but downregulated in Caucasians, and 25 showed the reverse pattern. Asian-specific upregulation of *AKR1B10*, *UBE2C*, and *S100P* suggests links to viral etiology and immune modulation, while *MDK*, *LCN2*, and *NQO1* were upregulated in Caucasians, implicating proliferative and metabolic roles. Functional enrichment analysis revealed distinct immune and metabolic pathways. Asians showed elevated ubiquitin ligase activity and suppressed inflammatory responses, while Caucasians exhibited enhanced cytokine signaling, complement activation, and xenobiotic metabolism.

**Conclusions:**

These findings highlight key molecular differences in HCC across ethnicities and emphasize the value of TCGA data for identifying both shared targets and population-specific therapeutic strategies. Understanding these differences is crucial for advancing precision oncology and developing tailored interventions.

## Introduction

Hepatocellular carcinoma (HCC), the most common form of primary liver cancer, remains one of the leading causes of cancer-related mortality worldwide [1]. Globally, liver cancer ranks as the sixth most common cancer and the third in mortality, reflecting its high fatality rate [2]. While advances have been made in understanding HCC’s molecular landscape, population-specific differences—particularly in immune responses and tumor microenvironment characteristics—are still insufficiently explored. Notably, disparities in HCC incidence, progression, and outcomes have been reported between Asian and Caucasian populations [3, 4].

Asia bears a disproportionate burden of HCC, accounting for over 70% of global cases, largely due to the high prevalence of chronic hepatitis B virus (HBV) and hepatitis C virus (HCV) infections [4, 5]. These infections promote immune dysregulation, persistent inflammation, and the development of an immunosuppressive tumor microenvironment [4–6]. In contrast, HCC in Caucasian populations, especially in Western countries, is more often linked to non-viral etiologies such as metabolic dysfunction-associated steatotic liver disease (MASLD) and alcohol-related liver disease [4]. These divergent etiologies contribute to distinct immunological landscapes and tumor biology, shaped by both genetic and environmental factors [5, 6].

Despite these epidemiological patterns, comparative molecular studies examining gene expression differences between Asian and Caucasian HCC patients remain limited [3, 5, 7]. Although socioeconomic factors contribute to racial health disparities, accumulating evidence suggests that genetic variations also influence cancer susceptibility and outcomes [7, 8]. For instance, differential mutation frequencies in key oncogenes and tumor suppressors, such as *TP53* and *RB1*, as well as distinct immune microenvironment profiles, have been observed across populations, impacting prognosis and treatment response [8, 9].

Leveraging The Cancer Genome Atlas (TCGA) dataset using the Liver Hepatocellular Carcinoma (LIHC) cohort [10, 11], which provides comprehensive transcriptomic data, this study investigates population-specific gene expression patterns in HCC. By identifying shared and ethnicity-specific molecular signatures, this research aims to enhance understanding of HCC pathogenesis and inform the development of more effective, population-tailored therapeutic strategies, contributing to improved clinical outcomes and reduced global disparities in liver cancer care.

## Materials and methods

### Data acquisition

Gene expression data for HCC and non-tumoral samples, along with clinical information for patients of Caucasian and Asian descent, were obtained from the TCGA-LIHC cohort (https://portal.gdc.cancer.gov/) [10, 11]. The dataset was obtained through open-access data, with demographic filters applied to select patients based on their self-reported ethnicity, as recorded in the clinical metadata, specifically focusing on Caucasian and Asian populations. All data were downloaded and merged into a raw count table in CSV format.

### Differential gene expression analysis

To identify differentially expressed genes (DEGs) between Caucasian and Asian HCC, the PyDESeq2 implementation of DESeq2 was utilized (https://pydeseq2.readthedocs.io/en/latest/). A DeseqDataSet object was created using the filtered count matrix and corresponding metadata. The design matrix was set to compare the “Caucasian” and “Asian” groups. For normalization, DESeq2 uses a method called size factor normalization to account for differences in sequencing depth between samples [12]. This step adjusts raw counts by dividing each sampleʼs counts by a sample-specific size factor, which ensures that comparisons across samples are not biased by library size.

### Dispersion estimation, differential expression thresholding, and gene analysis

Dispersion estimates were calculated for the data to model the gene-specific variance. The results were extracted using the DeseqStats function, which provided fold changes (FCs) and adjusted *p*-values of false discovery rate (FDR) for each gene. Genes with FDR < 0.05 and |log_2_FC| ≥ 1.0 were considered significantly differentially expressed. Genes with log_2_FC ≥ 1.0 were labelled upregulated, and those with log_2_FC ≤ −1.0 were labelled downregulated. These genes were retained for further analysis. Log_2_FC values were computed, and genes with log_2_FC ≥ 1.0 or ≤ –1.0 were classified as significantly upregulated or downregulated, respectively, and included in downstream analyses.

The analysis pipeline was implemented in a Jupyter Notebook environment (https://jupyter.org/) using Python packages including pandas  (https://pandas.pydata.org/), NumPy  (http://numpy.org/), Matplotlib  (https://matplotlib.org/), and seaborn  (https://seaborn.pydata.org/). All procedures were conducted with an emphasis on reproducibility and transparency in gene selection and data visualization.

### Gene annotation and visualization

To facilitate biological interpretation, gene symbols were mapped using the Sanbomics package (https://github.com/mousepixels/sanbomics). The Ensembl gene identifiers in the DESeq2 results were converted to human gene symbols based on mappings provided by the package using id_map tools.

Visualization of expression trends was carried out using the seaborn library (https://seaborn.pydata.org/). Heatmaps were generated to represent the log_2_FC values of the conserved genes. Annotated heatmaps were produced using a diverging color scale centered at zero to distinguish upregulated and downregulated patterns clearly.

### Survival analysis

The data of the TCGA-LIHC and the Genotype-Tissue Expression (GTEx) (https://www.gtexportal.org/home/) portals [10, 11, 13] were also analyzed and visualized by the GEPIA online tool [14]. Several biomarkers previously reported to play roles in HCC, including glypican-3 [15], Plasmalemmal Vesicle Associated Protein (PLVAP) [16], ficolin-3 [17], and OIT3 [18], were also considered in the interpretation of expression results. The prognostic relevance of DEGs on overall survival was evaluated using validated data from the Human Protein Atlas (HPA) (https://www.proteinatlas.org/) that uses the TCGA-LIHC cohort [19]. Patients were classified into two groups (high and low expression) based on the best expression cut-off, which refers to the fragments per kilobase of transcript per million mapped reads (FPKM) value that yields the maximal difference with regard to survival at the lowest log-rank *p*-value.

### Gene set enrichment analysis (GSEA)

To gain insights into biological processes (BPs) enriched in the DEGs, GSEA was performed using the GSEApy package (https://gseapy.readthedocs.io/en/latest/introduction.html). The gene rankings based on DESeq2 statistics were used as input. The analysis focused on Gene Ontology (GO) terms, including BPs, cellular components (CCs), and molecular functions (MFs). The enrichment results were visualized using bubble plots.

## Results

### Samples characteristic

The HCC cohort from the TCGA-LIHC database included 184 Caucasian and 157 Asian subjects, who were predominantly male. Notably, the Asian group had a markedly higher proportion of male subjects, approximately three times more than females. The mean age was significantly different between the two groups, 63.5 ± 13.9 years for Caucasians and 55 ± 13.9 years for Asians (*p* = 0.0001). Additionally, most of the Caucasian subjects were elderly patients (71.19%), whereas the Asian cohort had a younger age distribution. As controls, 34 non-tumoral samples were included for the Caucasian cohort and 6 for the Asian cohort. Both cohorts included patients with HCC ranging from stage I to stage IV, with the majority diagnosed at an early stage (stage I).

### Common DEGs in Asian and Caucasian LIHC populations compared to non-tumoral tissues

**Figure 1:**
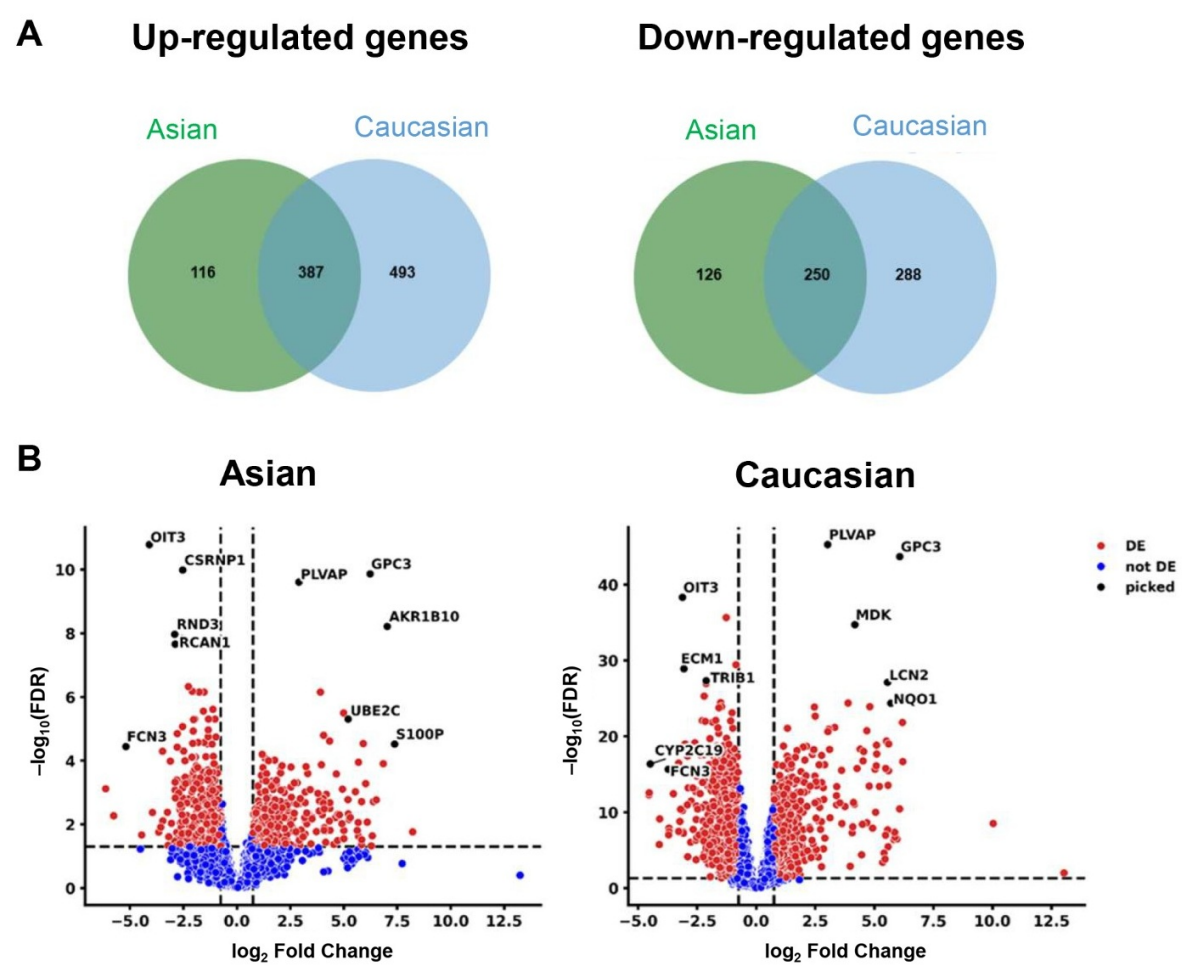
**Differentially expressed genes (DEGs) in Asian and Caucasian LIHC cohorts compared to non-tumoral tissues.** (**A**) Venn diagrams of upregulated and downregulated genes compared to non-tumoral tissues. (**B**) Volcano plots of downregulated and upregulated genes in hepatocellular carcinoma (HCC) in Asian [left panel] and in Caucasian [right panel] populations, compared to non-tumoral samples. FDR: false discovery rate; DE: differentially expressed.

Compared to non-tumoral tissues, a total of 503 genes were upregulated in the Asian LIHC and 880 genes in the Caucasian LIHC populations, of which 387 genes were upregulated in both populations (Figure 1A, Table S1). Among them, 116 genes were uniquely upregulated in Asians, while 493 were exclusive to Caucasians. In both groups, 250 genes were commonly downregulated; additionally, 126 genes were uniquely downregulated in Asians, and 288 in Caucasians (Figure 1A, Table S2). The full set of the DEGs in both populations—potentially representing shared molecular markers of HCC—is provided in Table S3.

Several genes were consistently dysregulated in both LIHC populations compared to the non-tumoral samples (Figure 1B). Notably, *GPC3* and *PLVAP* were upregulated, while *FCN3* and *OIT3* were downregulated in both the Asian and Caucasian populations. In contrast, distinct sets of genes exhibited population-specific expression patterns. In the Asian population, genes such as *AKR1B10, UBE2C,* and *S100P* were significantly upregulated, while *CSRNP1, RND3,* and *RCAN1* were significantly downregulated. Meanwhile, in the Caucasian cohort, *MDK, LCN2,* and *NQO1* were significantly upregulated, whereas *ECM1, TRIB1,* and *CYP2C19* were significantly downregulated.

### Prognostic relevance of common DEGs in both populations

**Figure 2:**
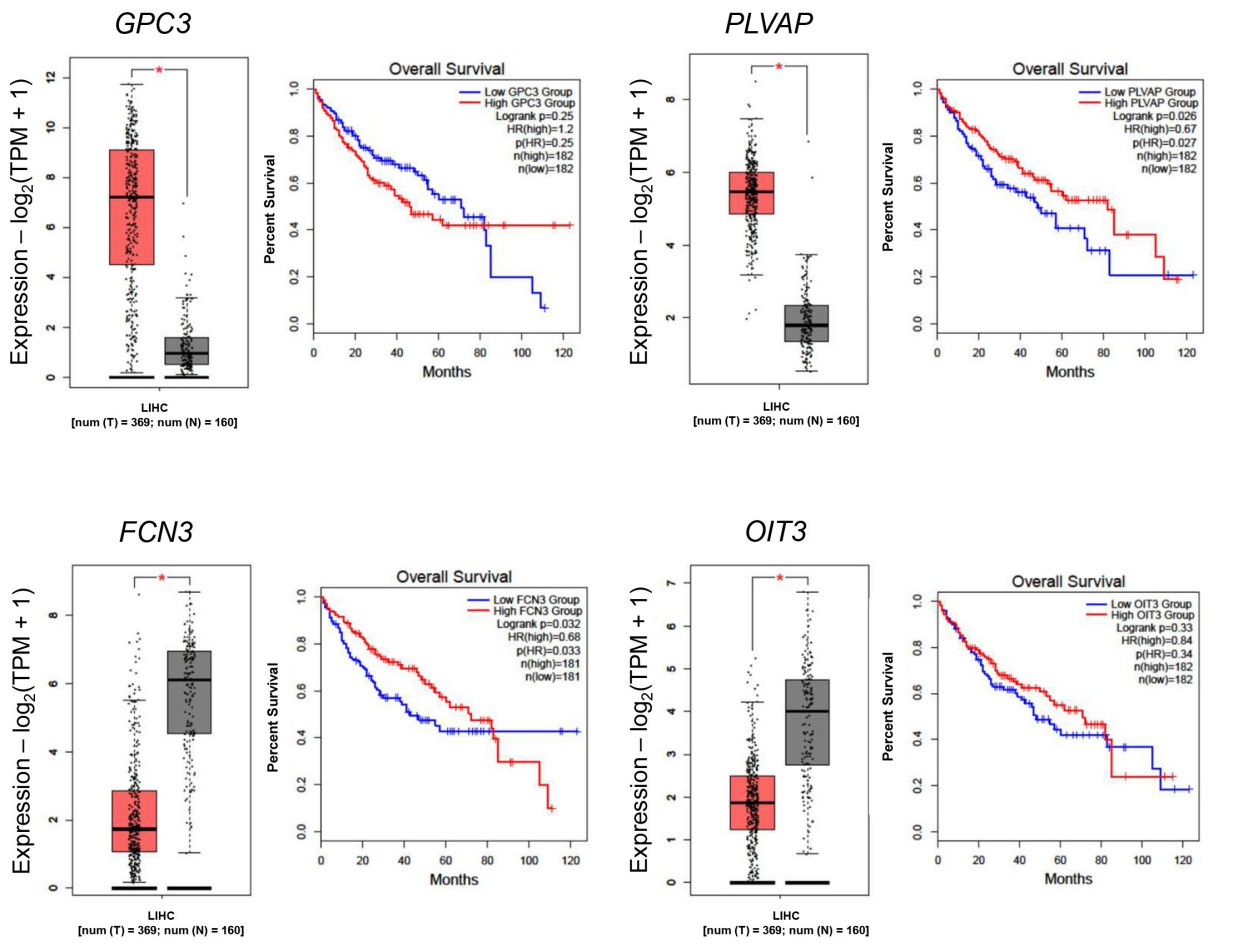
**Differential gene expression in the TCGA cohort and its association with overall survival in Asian and Caucasian cohorts.** Boxplot comparing the expression levels of consistently dysregulated genes in both cohorts. Red box: tumor tissue, grey box: normal tissue. *: *p* < 0.05.

Using GEPIA2 (http://gepia2.cancer-pku.cn/), the expression levels of these genes were obtained (Figure 2). Based on the HPA, the 5-year survival of patients with high *GPC3* expression was 46% against 60% in low-expressing patients, whereas patients with high *PLVAP* expression showed 54% versus 46% survival probability. Poorer prognosis was seen in patients with low *FCN3* expression (37% vs. 55% in high-expressing patients) and with low *OIT3* expression (42% vs. 63% in high-expressing patients).

**Figure 3:**
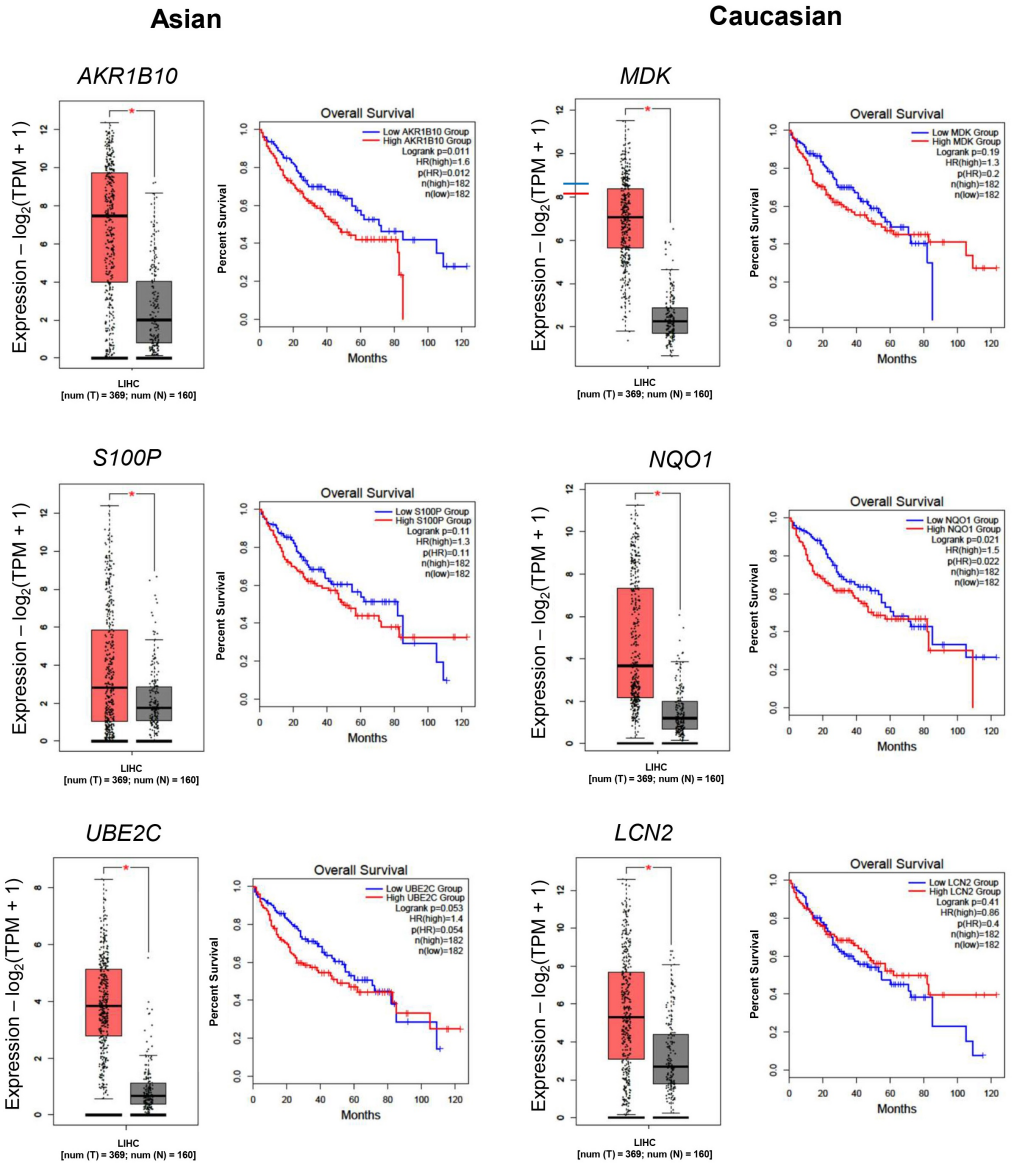
**Differential gene expressions in the TCGA cohort and its association with overall survival.** Boxplot comparing the expression levels of Asian-specific upregulated genes (*AKR1B10*, *S100P*, and *UBE2C*) and Caucasian-specific upregulated genes (*MDK*, *NQO1*, and *LCN2*). TPM: transcripts per million; LIHC: Liver Hepatocellular Carcinoma. Red box: tumor tissue, grey box: normal tissue. *: *p* < 0.05.

The expression of these genes in the TCGA-LIHC cohort, as well as the survival curves, is presented in Figure 3. Of particular interest is *AKR1B10,* which is distinctly upregulated in the Asian cohort, in which the 5-year survival probability in high-expressing patients was only 43% versus 58% in low-expressing patients. Overall, the high expression of genes in the Asian cohort resulted in a lower 5-year survival probability. In the Caucasian cohort, high *MDK* showed a poor 5-year survival rate of 38% versus 52% in low *MDK* patients. The same trend was observed for *NQO1* (39% in high-expressing versus 52% in low-expressing) and for *LCN2* (43% versus 51% in low-expressing).

To further explore the difference between the two groups, gene expression profiles between Caucasian and Asian cancer samples were compared using each population alternately as a reference. This analysis revealed several genes that were differentially expressed depending on the direction of comparison.

**Figure 4:**
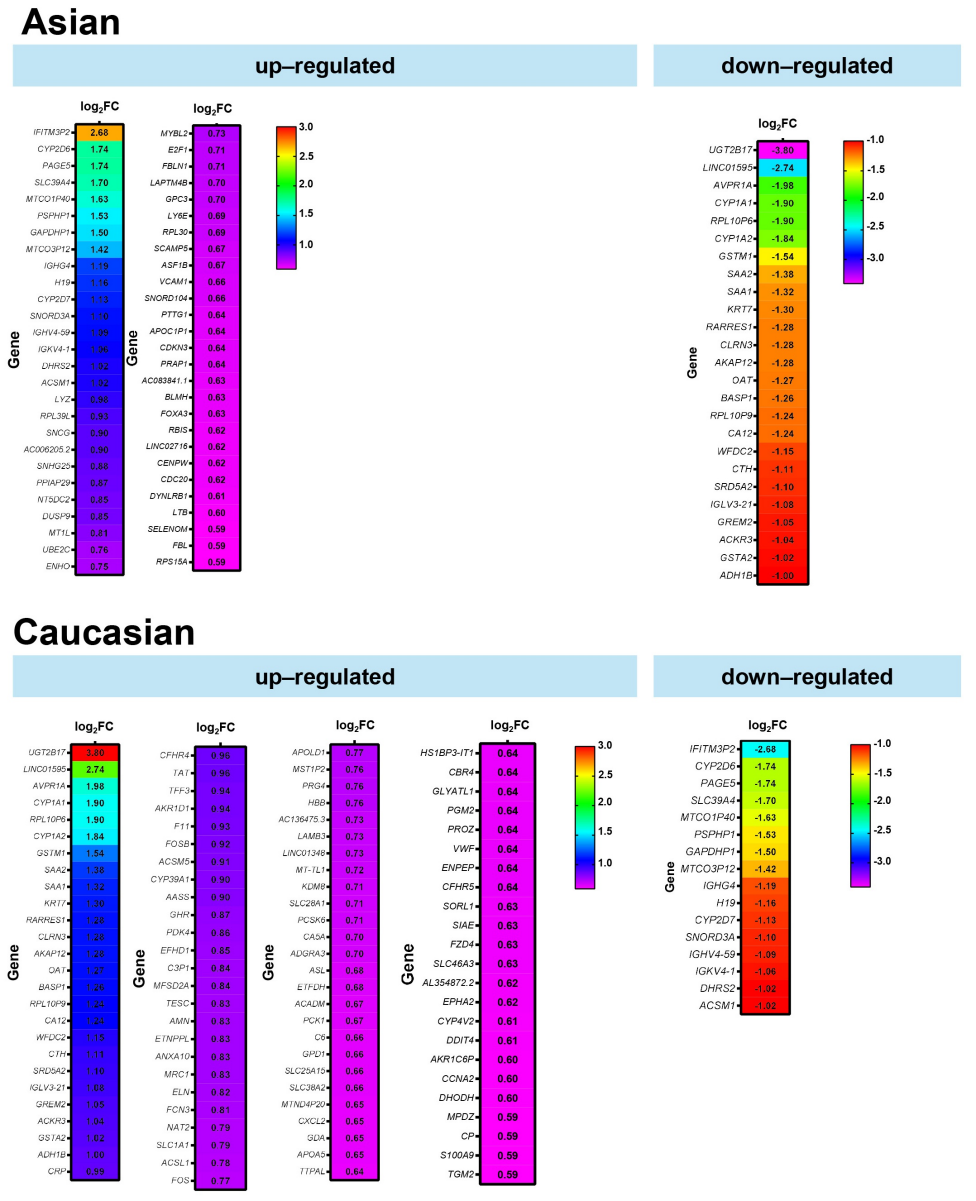
**Differentially expressed genes between Asian and Caucasian HCC patients.** Heatmaps displaying significantly upregulated and downregulated genes in Asian (top panel) and Caucasian (bottom panel) cohorts, based on RNA-seq data from the TCGA-LIHC dataset. The color scale represents the magnitude of log_2_FC. FC: fold change; HCC: hepatocellular carcinoma; TCGA: The Cancer Genome Atlas; LIHC: Liver Hepatocellular Carcinoma.

To complement the transcriptomic findings from the gene expression analysis, Figure 4 presents a list of genes that are differentially expressed in Asian and Caucasian cohorts. Heatmap analysis of DEGs revealed distinct transcriptional profiles in each of the Asian and Caucasian HCC populations. In the Asian population, 54 genes were upregulated compared to Caucasians, with *IFITM3P2* being the most highly upregulated gene with a log_2_FC of around 2.7, followed by *CYP2D6* and *PAGE5*. In contrast, 25 downregulated genes included *UGT2B17* (−3.80 log_2_FC), *LINC01595*, and *AVPR1A*, suggesting suppressed metabolic and regulatory pathways in the Asian group.

When comparing gene expression between the Caucasian and Asian cohorts, 100 genes showed higher expression in the Caucasian group, with *UGT2B17* (log_2_FC = 3.80), *LINC01595*, and *AVPR1A* standing out as the most upregulated. These genes are associated with increased xenobiotic metabolism and immune regulatory activity. Meanwhile, 16 genes, such as *IFITM3P2*, were significantly downregulated. The consistent *q*-value distribution across both populations supports the reliability of these findings.

**Figure 5:**
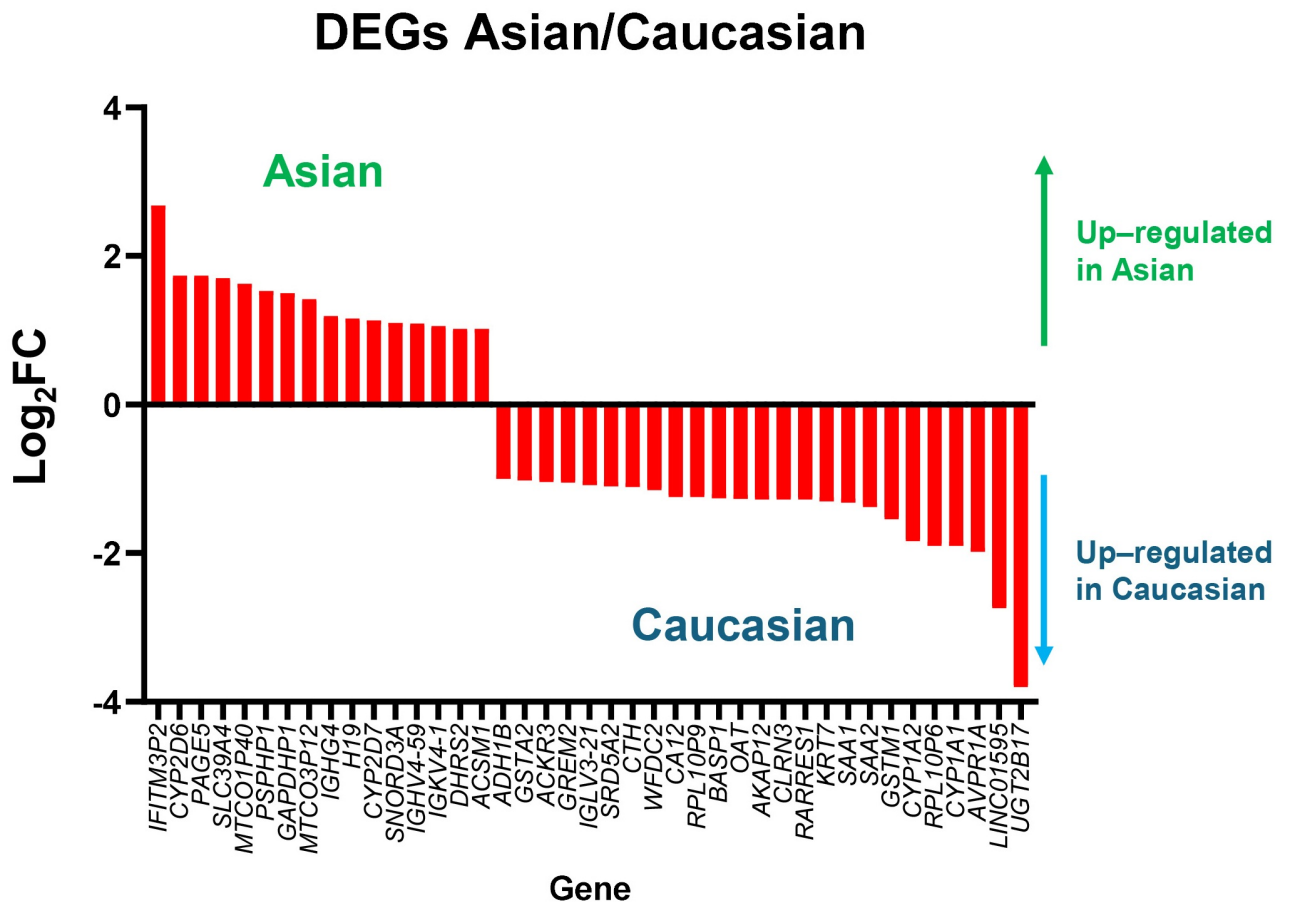
**Genes with opposite expression patterns in Asian and Caucasian HCC patients.** Bar plot showing log_2_ fold changes of genes that are upregulated in one population but downregulated in the other, based on TCGA-LIHC data. Positive values indicate higher expression in Asians, and negative values indicate higher expression in Caucasians. DEGs: differentially expressed genes; FC: fold change; HCC: hepatocellular carcinoma; TCGA: The Cancer Genome Atlas; LIHC: Liver Hepatocellular Carcinoma.

To simplify Figure 4, differential gene expression analysis between Asian and Caucasian HCC samples identified a distinct subset of genes with opposing expression patterns across the two populations, as shown in Figure 5. A total of 16 genes were significantly upregulated in the Asian cohort but downregulated in the Caucasian group, while 25 genes showed the reverse trend—upregulated in Caucasians but downregulated in Asians. The bar plot illustrates the log_2_FC of these genes, with positive values indicating higher expression in Asians and negative values indicating higher expression in Caucasians. Among the 16 genes upregulated in Asians, *IFITM3P2* had more than 2.5-fold higher expression in Asians compared to Caucasians. Conversely, among the 25 genes downregulated in Asians but upregulated in Caucasians, *UGT2B17* and *LINC01595* showed over 2.5-fold higher expression in Caucasians.

### Enriched BPs associated with the alteration of gene expression

Both groups underwent GO BP enrichment analysis to gain a better understanding of the shared functional impact of upregulated and downregulated genes between the two populations. The identification of the biological pathways and functional roles associated with the DEGs highlighted key BPs in each group. The enriched BP, MF, and CC keywords for commonly upregulated and downregulated genes are shown in Tables S4 and S5, respectively.

### Functional enrichment analysis of the DEGs

**Figure 6:**
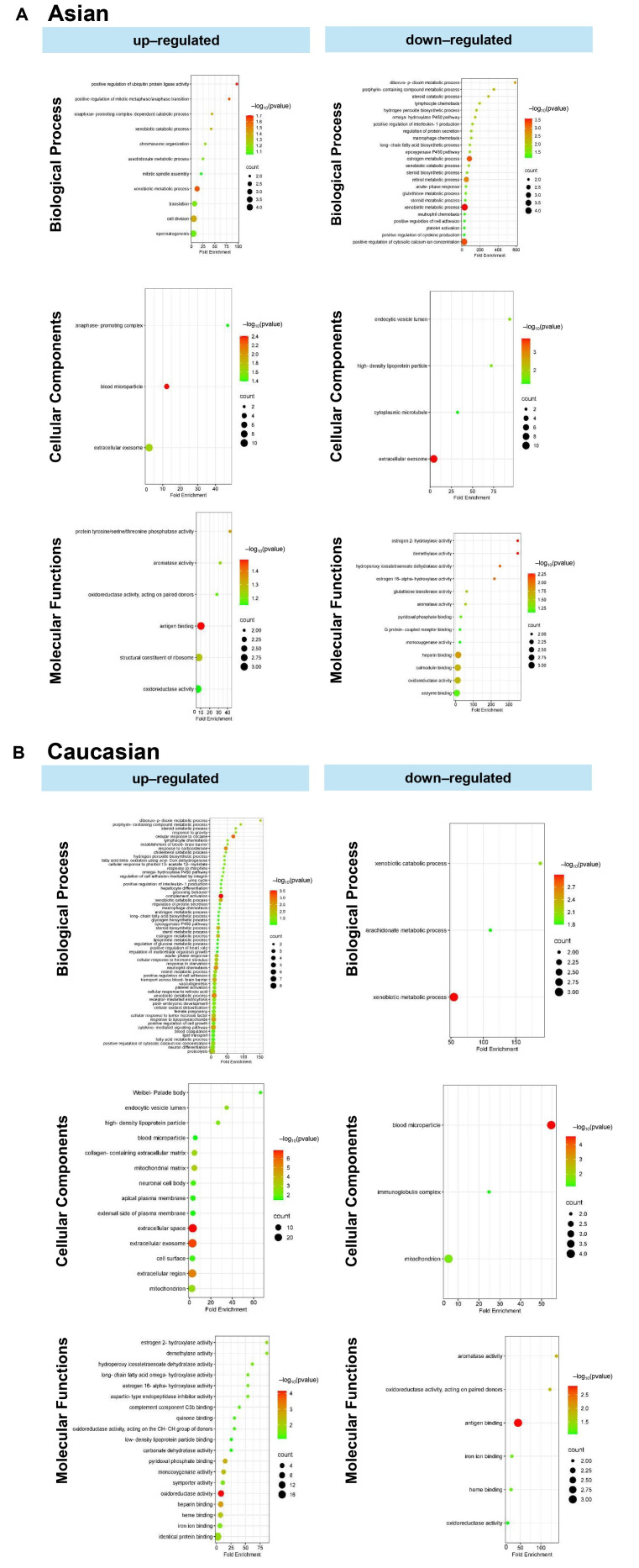
**Gene ontology analysis comprises biological processes, cellular components, and molecular functions.** (**A**) Unique upregulated and downregulated genes in the Asian population. (**B**) Unique upregulated and downregulated genes in the Caucasian population.

To identify population-specific molecular signatures in HCC, functional enrichment analysis was performed on DEGs between the Asian and Caucasian populations. In the Asian population, upregulated genes were significantly enriched in BP, such as positive regulation of ubiquitin protein ligase activity, mitotic metaphase/anaphase transition, and anaphase-promoting complex-dependent catabolic processes (Figure 6A). Enrichment in CC included blood microparticles, extracellular exosomes, and the anaphase-promoting complex, while MF terms involved antigen binding, structural constituents of ribosome, and protein tyrosine/serine/threonine phosphatase activity. Conversely, downregulated genes in the Asian population were associated with BP, including dibenzo-p-dioxin metabolic process, porphyrin-containing compound metabolism, steroid catabolic process, and various chemotactic responses such as lymphocyte and macrophage chemotaxis. These genes were also enriched in CC, such as high-density lipoprotein particles, endocytic vesicles, and cytoplasmic microtubules. In terms of MF, significant reductions were observed in activities related to estrogen hydroxylase, glutathione transferase, and enzyme binding.

In the analysis of upregulated genes in the Caucasian population (Figure 6B), several BP were significantly enriched, including positive regulation of interleukin-1β secretion, macrophage and neutrophil migration, cytokine production, dibenzo-p-dioxin and porphyrin-containing compound metabolism, peroxide catabolism, and positive regulation of cell adhesion molecules. At the cellular level, these genes were predominantly associated with the extracellular space, exosomes, and the cell surface. Additional enrichment in mitochondrial and plasma membrane components highlights the interplay between energy metabolism, vesicle trafficking, and membrane-associated signaling in HCC progression. MF analysis revealed dysregulation in redox homeostasis and hormone metabolism, with significant enrichment in oxidoreductase and hydroxylase activities, particularly those involved in estrogen and fatty acid metabolism. Binding activities related to heme, iron ions, and cofactors were also prominently enriched.

Conversely, downregulated genes in the Caucasian population were enriched in pathways related to xenobiotic metabolism, xenobiotic catabolism, and arachidonate metabolism. At the cellular level, these genes were associated with blood microparticles, the immunoglobulin complex, and mitochondria. MF analysis indicated suppression of key activities, such as antigen binding, aromatase activity, oxidoreductase activity, and heme/iron ion binding.

Kyoto Encyclopedia of Genes and Genomes (KEGG) enrichment analyses revealed distinct pathway signatures between ethnic cohorts and controls (Figures S1–4). In the Asian cohort compared with controls, the cell cycle pathway was the most significantly enriched (FDR = 7.2 × 10^–5^), characterized by a general upregulation of cyclin-cyclin-dependent kinase (cyclin-CDK) complexes and checkpoint regulators, including the anaphase-promoting complex/cyclosome (APC/C) ubiquitin-mediated proteolysis system, suggesting a strong proliferative drive. In the Caucasian cohort, the cell cycle pathway was also enriched, though more modestly (FDR = 0.076), with dysregulation predominantly involving CDK1/CDK2 and APC/C.

Cross-ethnic comparisons further highlighted divergent patterns. In the Asian tumors, strong enrichment was observed in xenobiotic metabolism and chemical carcinogenesis pathways (FDR = 2.0 × 10^–6^), driven by cytochrome P450 enzymes and DNA adduct repair genes, consistent with processes related to alcohol metabolism and detoxification of environmental carcinogens (Figure S4). Conversely, the Asian-up vs. Caucasian analysis revealed enrichment in endocrine resistance (FDR = 0.15), implicating estrogen receptor signaling and activation of the PI3K/AKT pathway.

## Discussion

This study reveals both shared and population-specific gene expression patterns in HCC between Asian and Caucasian populations. A total of 387 and 250 genes were commonly upregulated and downregulated, respectively, in both groups (Figure 1A), suggesting conserved molecular mechanisms underlying HCC pathogenesis. Among these, *GPC3* and *PLVAP* were consistently upregulated, implicating their roles in tumor progression and angiogenesis across ethnicities (Figure 1B) [15, 16]. In contrast, *FCN3* and *OIT3* were commonly downregulated, both known for their tumor-suppressive and immune-regulatory functions, highlighting their potential as universal biomarkers or therapeutic targets [17, 18]. These data were then validated in the tissue distribution and patients’ prognosis (Figures 2 and 3).

Distinct gene expression signatures were also observed between the two populations (Figures 4 and 5). In the Asian population, upregulated genes such as *AKR1B10*, *UBE2C*, and *S100P* are involved in immune modulation and tumor progression [19–23]. Conversely, *CSRNP1*, *RND3*, and *RCAN1* were significantly downregulated, each linked to anti-proliferative or prognostic roles in cancer [24–26]. These differences may reflect the influence of HBV/HCV-driven immune responses more prevalent in Asian populations [20, 21]. Consistently, a study comparing Vietnamese and Italian HCC cohorts demonstrated distinct correlations of cancer stem cell marker CD90/THY-1 in HBV-related HCC, with stronger associations observed in Vietnamese patients (Asian) compared to Italian patients (Caucasian) [27].

In the Caucasian population, *MDK*, *LCN2*, and *NQO1* were significantly upregulated, indicating enhanced oncogenic and metabolic activity [28–30]. Meanwhile, *ECM1*, *TRIB1*, and *CYP2C19* were downregulated, implicating impaired liver fibrosis regulation, carcinogen metabolism, and potential disruption of key signaling pathways [31–33].

Importantly, 16 genes were found to be upregulated in Asians but downregulated in Caucasians, and 25 showed the opposite pattern. *IFITM3P2*, upregulated over 2.5-fold in Asians, may contribute to antiviral immune responses and influence HCC susceptibility, especially in HBV/HCV-endemic regions [34, 35]. In contrast, *UGT2B17* and *LINC01595* were markedly downregulated in Asians but upregulated in Caucasians, with *UGT2B17* deficiency linked to disrupted metabolism and heightened immune activation [36].

These population-specific gene signatures are likely influenced by differing HCC etiologies—viral infections in Asians and metabolic syndromes in Caucasians [35, 37]. Such variation underscores the need for personalized approaches to HCC treatment based on genetic and ethnic context.

GO enrichment analysis further revealed immune-regulatory differences between groups (Figure 6). In Asians, upregulated genes were associated with ubiquitin ligase activity and mitotic regulation, suggesting enhanced immune evasion and tumor proliferation [38]. Elevated antigen binding and phosphatase activity also indicated altered immune signaling [39]. However, genes involved in inflammatory responses—such as interleukin-1 production and chemotaxis—were downregulated, pointing to a suppressed inflammatory environment. In addition, metabolic pathways, including steroid and glutathione metabolism, were reduced, suggesting impaired immune-metabolic crosstalk [38–40].

Conversely, Caucasian populations exhibited enrichment of genes involved in complement activation, cytokine signaling, and neutrophil chemotaxis, indicating a heightened pro-inflammatory and immune-active state [7]. Metabolic pathways such as steroid biosynthesis, retinol metabolism, and xenobiotic catabolism were also more active, possibly reflecting greater involvement of metabolism-associated HCC in this group [41]. MFs such as oxidoreductase and estrogen hydroxylation were significantly enriched, highlighting distinct metabolic adaptations in Caucasian populations [41, 42].

Pathway-level analysis highlights both shared and population-specific mechanisms of tumor biology (Figures S1–4). Dysregulation of cell cycle control emerged as a common feature, but it was more pronounced and widespread in the Asian subset, involving cyclins, checkpoint regulators, and the ubiquitin-mediated proteolysis pathway. By contrast, pathways related to xenobiotic metabolism and chemical carcinogenesis were strongly enriched in the Caucasian subset, reflecting a molecular signature consistent with alcohol exposure and environmental carcinogen burden—findings that align with epidemiological data linking alcohol consumption to increased cancer risk.

Conversely, the Asian cohort showed enrichment for endocrine resistance, suggesting distinct patterns of hormone receptor signaling and survival-related pathways. Taken together, while both populations exhibit loss of proliferative control, the molecular context may differ: Caucasians appear more vulnerable through environmentally and metabolically driven mechanisms, whereas Asians display a more pronounced dysregulation involving endocrine signaling.

Together, these immunological and metabolic differences may help explain variation in disease progression, treatment response, and survival outcomes between ethnic groups. Understanding population-specific gene dysregulation may provide valuable insights into liver cancer pathogenesis and support the development of targeted and equitable therapeutic strategies. A key strength of this study lies in its identification of both commonly dysregulated genes and population-specific expression patterns, offering potential biomarkers for universal and tailored HCC management. However, limitations include the inherent genetic and cultural heterogeneity within racial groups and the lack of detailed clinical metadata. In this study, we did not stratify or adjust the analyses according to clinical etiologies such as HBV/HCV serology, MASLD, or alcohol consumption. The primary scope was to characterize the racial background of the patients in relation to the most significant DEGs and to explore whether molecular patterns could be detected across racial groups irrespective of underlying etiology, while recognizing that etiology-specific differences may play an important role. Another important direction will be the incorporation of the genomic data in the TCGA, such as comprehensive copy number variation (CNV) and somatic mutations. CNV and somatic mutations will surely provide a more comprehensive understanding of whether transcriptional differences between ethnic cohorts align with underlying genomic alterations. Future work with larger and more comprehensively annotated cohorts will be needed to perform stratified analyses that directly evaluate the combined impact of etiology and racial background on gene expression patterns.

### Conclusion

This study reveals significant ethnic-specific differences in gene expression, immune responses, and metabolic pathways between Asian and Caucasian liver cancer patients, highlighting distinct molecular mechanisms underlying HCC pathogenesis. These findings underscore the need for population-specific therapeutic strategies and support the integration of molecular profiling into precision medicine to improve treatment outcomes across diverse patient groups. Future research should focus on translating these molecular insights into targeted immunotherapies and personalized treatment approaches to enhance clinical efficacy and reduce health disparities in liver cancer care.

## Abbreviations

APC/C: anaphase-promoting complex/cyclosome
BPs: biological processes
CCs: cellular components
CDK: cyclin-dependent kinase
CNV: copy number variation
DEGs: differentially expressed genes
FCs: fold changes
FDR: false discovery rate
GO: Gene Ontology
GSEA: gene set enrichment analysis
HBV: hepatitis B virus
HCC: hepatocellular carcinoma
HCV: hepatitis C virus
HPA: Human Protein Atlas
LIHC: Liver Hepatocellular Carcinoma
MASLD: metabolic dysfunction-associated steatotic liver disease
MFs: molecular functions
TCGA: The Cancer Genome Atlas

## Supplementary materials

The supplementary tables for this article are available at: https://www.explorationpub.com/uploads/Article/file/1002344_sup_1.pdf. The supplementary figures for this article are available at: https://www.explorationpub.com/uploads/Article/file/1002344_sup_2.pdf.

## Declarations

### Availability of data and materials

The data that support the findings of this study are available on reasonable request from the corresponding author.

### Copyright

© The Author(s) 2025.

## References

[1] Kim DY (2024). Changing etiology and epidemiology of hepatocellular carcinoma: Asia and worldwide. J Liver Cancer.

[2] Bray F, Laversanne M, Sung H, Ferlay J, Siegel RL, Soerjomataram I (2024). Global cancer statistics 2022: GLOBOCAN estimates of incidence and mortality worldwide for 36 cancers in 185 countries. CA Cancer J Clin.

[3] GBD US Health Disparities Collaborators (2024). Burden of liver cancer mortality by county, race, and ethnicity in the USA, 2000-19: a systematic analysis of health disparities. Lancet Public Health.

[4] Samant H, Amiri HS, Zibari GB (2021). Addressing the worldwide hepatocellular carcinoma: epidemiology, prevention and management. J Gastrointest Oncol.

[5] Kim NJ, Cravero A, VoPham T, Vutien P, Carr R, Issaka RB (2023). Addressing racial and ethnic disparities in US liver cancer care. Hepatol Commun.

[6] Vidal AC, Moylan CA, Wilder J, Grant DJ, Murphy SK, Hoyo C (2022). Racial disparities in liver cancer: Evidence for a role of environmental contaminants and the epigenome. Front Oncol.

[7] Varghese RS, Barefoot ME, Jain S, Chen Y, Zhang Y, Alley A (2021). Integrative Analysis of DNA Methylation and microRNA Expression Reveals Mechanisms of Racial Heterogeneity in Hepatocellular Carcinoma. Front Genet.

[8] Duan X, Cai Y, He T, Shi X, Zhao J, Zhang H (2021). The effect of the *TP53* and *RB1* mutations on the survival of hepatocellular carcinoma patients with different racial backgrounds. J Gastrointest Oncol.

[9] Wen GM, Song CL, Liu DH, Xia P (2023). Different races have different immune microenvironments: comparison of White and Asian patients with liver cancer. Am J Cancer Res.

[10] Weinstein JN, Collisson EA, Mills GB, Shaw KR, Ozenberger BA, Ellrott K, Cancer Genome Atlas Research Network (2013). The Cancer Genome Atlas Pan-Cancer analysis project. Nat Genet.

[11] (2017). Cancer Genome Atlas Research Network. Comprehensive and Integrative Genomic Characterization of Hepatocellular Carcinoma. Cell.

[12] Love MI, Huber W, Anders S (2014). Moderated estimation of fold change and dispersion for RNA-seq data with DESeq2. Genome Biol.

[13] Ardlie KG, Deluca DS, Segrè AV, Sullivan TJ, Young TR, Gelfand ET, The GTEx Consortium (2015). Human genomics. The Genotype-Tissue Expression (GTEx) pilot analysis: multitissue gene regulation in humans. Science.

[14] Tang Z, Li C, Kang B, Gao G, Li C, Zhang Z (2017). GEPIA: a web server for cancer and normal gene expression profiling and interactive analyses. Nucleic Acids Res.

[15] Zhou F, Shang W, Yu X, Tian J (2018). Glypican-3: A promising biomarker for hepatocellular carcinoma diagnosis and treatment. Med Res Rev.

[16] Wang Y, Cheng T, Chen T, Chang K, Chuang VP, Kao K (2014). Plasmalemmal Vesicle Associated Protein (PLVAP) as a therapeutic target for treatment of hepatocellular carcinoma. BMC Cancer.

[17] Zheng G, Wu L, Bouamar H, Cserhati M, Chiu Y, Hinck CS (2024). Ficolin-3 induces apoptosis and suppresses malignant property of hepatocellular carcinoma cells via the complement pathway. Life Sci.

[18] Yang S, Zhang J, Xu Y, Wang J, Zhao H, Lei J (2022). OIT3 mediates macrophage polarization and facilitates hepatocellular carcinoma progression. Cancer Immunol Immunother.

[19] Wu A, Li H, Gao M, Liang J, Huang J, Farrés J (2024). The pan-cancer landscape of aldo-keto reductase1B10 reveals that its expression is diminished in gastric cancer. Front Immunol.

[20] Semmo N, Weber T, Idle JR, Beyoğlu D (2015). Metabolomics reveals that aldose reductase activity due to AKR1B10 is upregulated in hepatitis C virus infection. J Viral Hepat.

[21] Mori M, Genda T, Ichida T, Murata A, Kamei M, Tsuzura H (2017). Aldo-keto reductase family 1 member B10 is associated with hepatitis B virus-related hepatocellular carcinoma risk. Hepatol Res.

[22] Zhan P, Lu Y, Lu J, Cheng Y, Luo C, Yang F (2024). The activation of the Notch signaling pathway by UBE2C promotes the proliferation and metastasis of hepatocellular carcinoma. Sci Rep.

[23] Singh P, Ali SA (2022). Multifunctional Role of S100 Protein Family in the Immune System: An Update. Cells.

[24] Xu B, Lv W, Li X, Zhang L, Lin J (2019). Prognostic genes of hepatocellular carcinoma based on gene coexpression network analysis. J Cell Biochem.

[25] Basbous S, Paysan L, Sena S, Allain N, Hiriart J, Dugot-Senant N (2022). Silencing of *RND3/RHOE* inhibits the growth of human hepatocellular carcinoma and is associated with reversible senescence. Cancer Gene Ther.

[26] Yang Z, Deng X, Wen D, Sun L, An R, Xu J (2024). Identification of RCAN1ʼs role in hepatocellular carcinoma using single-cell analysis. BMC Cancer.

[27] Luong AB, Do HQ, Tarchi P, Bonazza D, Bottin C, Cabral LKD (2020). The mRNA Distribution of Cancer Stem Cell Marker CD90/Thy-1 Is Comparable in Hepatocellular Carcinoma of Eastern and Western Populations. Cells.

[28] Wang X, Liu Y, Han A, Tang C, Xu R, Feng L (2022). The NQO1/p53/SREBP1 axis promotes hepatocellular carcinoma progression and metastasis by regulating Snail stability. Oncogene.

[29] Christou C, Stylianou A, Gkretsi V (2024). Midkine (MDK) in Hepatocellular Carcinoma: More than a Biomarker. Cells.

[30] Han B, An Z, Gong T, Pu Y, Liu K (2024). LCN2 Promotes Proliferation and Glycolysis by Activating the JAK2/STAT3 Signaling Pathway in Hepatocellular Carcinoma. Appl Biochem Biotechnol.

[31] Ashida R, Okamura Y, Ohshima K, Kakuda Y, Uesaka K, Sugiura T (2018). The down-regulation of the CYP2C19 gene is associated with aggressive tumor potential and the poorer recurrence-free survival of hepatocellular carcinoma. Oncotarget.

[32] Link F, Li Y, Zhao J, Munker S, Fan W, Nwosu ZC (2025). ECM1 attenuates hepatic fibrosis by interfering with mediators of latent TGF-β1 activation. Gut.

[33] Ye Y, Wang G, Wang G, Zhuang J, He S, Song Y (2017). The Oncogenic Role of Tribbles 1 in Hepatocellular Carcinoma Is Mediated by a Feedback Loop Involving microRNA-23a and p53. Front Physiol.

[34] Huang L, Hsieh T, Huang C, Liu Y, Wu S, Chien P (2021). Disparity of Hepatocellular Carcinoma in Tumor Microenvironment-Related Genes and Infiltrating Immune Cells between Asian and Non-Asian Populations. Genes (Basel).

[35] Kawamura A, Matsuda K, Murakami Y, Saruta M, Kohno T, Shiraishi K (2023). Contribution of an Asian-prevalent HLA haplotype to the risk of HBV-related hepatocellular carcinoma. Sci Rep.

[36] Rouleau M, Schwab M, Klein K, Tremmel R, Haag M, Schaeffeler E (2025). The liver proteome of individuals with a natural UGT2B17 complete deficiency. Sci Rep.

[37] Kaya NA, Chen J, Lai H, Yang H, Ma L, Liu X (2022). Genome instability is associated with ethnic differences between Asians and Europeans in hepatocellular carcinoma. Theranostics.

[38] Chen K, Shuen TWH, Chow PKH (2024). The association between tumour heterogeneity and immune evasion mechanisms in hepatocellular carcinoma and its clinical implications. Br J Cancer.

[39] Zheng X, Yang L, Shen X, Pan J, Chen Y, Chen J (2024). Targeting *Gsk3a* reverses immune evasion to enhance immunotherapy in hepatocellular carcinoma. J Immunother Cancer.

[40] Chen C, Wang Z, Ding Y, Qin Y (2023). Tumor microenvironment-mediated immune evasion in hepatocellular carcinoma. Front Immunol.

[41] Khamis ZI, Pang X, Cui Z, Sang QA, Zhang J (2021). Cytochrome P450-2D6: A novel biomarker in liver cancer health disparity. PLoS One.

[42] Wu H, Liu Y, Liu Q, Li Z, Wan Y, Cao C (2024). HMMR triggers immune evasion of hepatocellular carcinoma by inactivation of phagocyte killing. Sci Adv.

