# Supplementary material for: Racial disparities in hepatocellular carcinoma: a TCGA-based gene expression study of Caucasian and Asian populations
Source: Explor Target Antitumor Ther. 2025 Nov 2;6:1002344. doi: 10.37349/etat.2025.1002344 (PMC12597399; doi:10.37349/etat.2025.1002344)
Supplement: Supplementary file 1 [file 1002344_sup_1.pdf]

Supplementary Tables

Table S1. List of upregulated unique genes for each population.

Population	Unique genes
Asian	*AARS2, AATF, AC005332.3, ACBD6, ADM2, AL365181.3, ALG1L, AP5Z1, ASF1B, ATIC, AURKB, BANF1, BLOC1S3, C1orf35, CAD, CASC3, CCL25, CCNB2, CD2BP2, CDCA8, CDK1, CDKN2A, CDKN3, CDT1, CENPM, CHAF1A, CHTOP, COPS7B, CSE1L, CTHRC1, DAXX, DCAF13, DEDD, DHX34, DKK1, DVL2, DYNLL1, EHMT2, EPS8L3, FAM222A, FBL, FGF21, FN3KRP, GLA, GSDMB, HDAC11, HES6, HGS, HNRNPA1, HSPA1A, HSPA1B, IGF2BP2, ITGB1BP1, ITPKA, KIF22, KIFC1, LIG1, LYSMD1, METTL18, MROH1, NAA20, NCAPD2, NF2, NMB, NPM1, NT5DC2, NUP62, P3H4, PABPC1L, PLPP2, POC1A, PPOX, PYCR1, RBIS, RBMX, RECQL4, RFC4, RHNO1, RNPEPL1, RNU6-8, RPL23A, RRP1, RUSC1, SCARA3, SLC26A6, SNAPIN, SNHG1, SNHG25, SNHG7, SP5, SPC24, SPSB2, SREBF2, SUV39H1, TACC3, TAFAZZIN, TARBP1, TBC1D16, TCF19, TEAD3, TJAP1, TMED9, TOMM20, TPRKB, TRIM65, TRMT2A, TTC13, TUBB, TUFT1, UBE2O, UBE2T, USP21, VPS45, WDR5, XPO5, ZBED10P*
Caucasian	*NFYA, PLXND1, SPPL2B, TMEM98, GGCT, RPUSD1, CYB561, IL32, MVP, XYLT2, BIRC3, EHD2, RRAGD, TYMP, BTN3A1, BAK1, TMSB10, AIFM2, ATP6V1H, TPR, TSPAN17, CYBA, ATP2B4, CS, SUGP2, ME1, SMARCD1, ATP1B3, UFD1, NUCB2, GBA2, ATP6AP1, TRIB2, RHOBTB1, XRCC1, TEAD2, CA12, RAP1GAP, NFKB2, SIRT6, UBE2A, FBLN1, SCGN, COL5A3, DLGAP4, ATP8B1, FAT1, BAX, DNM1L, PIR, EPB41L1, ARHGAP4, STRN4, GOLGA3, SEL1L3, TMEM38B, PGC, ERMP1, TSPAN15, WASHC2A, CEP170B, RASSF7, LGALS1, PIK3IP1, TRIOBP, KDELR3, RHBDD3, FOXRED2, ABHD4, ABHD12, SLC17A9, C20orf27, CDC25B, ARFRP1, HM13, PXMP4, WFDC2, PSMD10, WDR13, ABCD1, MAGED2, MAPK3, CLCN7, PIEZO1, PYCARD, NIPAL2, EEF1D, ANXA13, RELB, ARHGEF18, YJU2B, PPP6R1, SLC1A5, TGFB1, RPS19, PLPPR2, BCAT2, ARRDC2, ISYNA1, TMEM147, ATP13A1, PON2, ABHD11, GARS1, SNX8, MOGAT3, BLVRA, YKT6, CA9, ABCA2, RASSF4, LZTS2, NPM3, CPD, COL1A1, RAB34, CPE, WFS1, MFSD10, GALNT18, TCIRG1, VWF, MLEC, MGP, TIMELESS, CILK1, VNN2, SLC39A7, SLC29A1, HARS2, APBB3, SPARC, SUB1, SLC12A7, GNPDA1, PDGFRB, CCL20, MPV17, PSMD14, EFEMP1, REG1A, TIA1, FAM20B, SRM, FBXO2, P3H1, RARRES1, IFI27L2, EPCAM, NUP43, CYSTM1, PDZD11, HSPH1, COPS5, SCPEP1, DESI2, TMEM54, OCRL, PAEP, GIPC1, TUBA1B, LPGAT1, PIGT, USP22, MYRF, DOK4, RAP2A, SOX9, MIF4GD, PPDPF, SDCBP2, BCL2L12, SSX1, MAP2K2, TSPAN8, TUBA4A, SDF2L1, GAL3ST1, ATP6V1F, SMO, MRPS12, DUT, PALLD, AP1M2, RNASE1, TOMM40, COL5A1, LAMA5, HIP1R, RAMP2, PRKAB2, SNRPA1, LGALS3, RAMP1, TRIM47, RHBG, AP3B1, FBXO44, CHI3L1, NTS, PTGFRN, FKBP11, RTL8C, GOLM1, CD36, TES, CDK4, GLUL, BZW2, TTYH3, SAP130, UGGT1, TXN, DAB2IP, CDK5RAP2, DPM2, ENPP2, GGH, PREB, SLC5A6, ITGAV, CXCL9, PAPSS1, COMMD4, ULK3, CMTM3, NOL3, OSGIN1, DEF8, G6PC3, SECTM1, CBX4, FKBP10, HSPG2, SLC44A3, ATP1B1, POGK, COP1, MRPL9, POGZ, EIF2D, INTS7, SMYD2, INTS3, PARP1, CCNA2, DCDC2, SLC17A4, FAM193B, RNF44, GNA12, C7orf50, OGT, PLPPR1, GSN, CIZ1, GPR107, CYP17A1, ST14, HYOU1, TAOK2, CCT5, AKR1C2, TMEM45B, HSPB8, GJA1, CLGN, RASSF3, LPCAT1, LY96, BTG3, LARP1, BATF, ODR4, MMP14, WASF2, LAD1, CYB5R1, PSKH1, NPR2,*

	TFF3, PDXK, SHKBP1, ZBTB7B, SHC1, ANO10, PMF1, GPATCH4, FGFR4, NACC1, PPP1R16A, SQSTM1, DVL3, ITGA5, JOSD2, FLYWCH2, KLHL21, SELENON, BROX, PKDCC, S100A11, COL6A3, NAXE, ATP1A1, ANKZF1, PRKCD, FOXQ1, SLC29A4, COL1A2, CDK5, SLC4A2, FASTK, COX6C, WASHC5, SPTSSA, INPPL1, ZNF503, ZMYND19, NSMF, IFI27, SPINT1, CKB, TRMT61A, MARS1, PRR15L, RRM1, MVD, LENG8, YIF1B, MFSD3, CD320, CYP7A1, FTH1, TAP1, REEP4, ATXN2L, COL3A1, PCSK9, PTK2, LRRC45, CENPX, ASPSCR1, FASN, RAC3, FABP4, COX7B2, GSTA4, BBLN, LGALS4, PWWP2B, CYP4F22, MRPL13, BPGM, MAB21L4, HCFC1, CORO1B, LRRC20, EGFL7, ADCK5, MTX1, GPR137, PPP1R14B, RNF213, PHLDA3, SLC16A11, SLC29A2, BRMS1, CD248, PDZK1, CSRP2, DCTN2, PNMA1, DEAF1, ERICH5, CD151, TMEM94, SOX12, PLEC, MSC, APOLD1, EXOSC4, RRS1, RCC2, SAMD4B, B3GNT3, DCTPP1, PPP1R14BP3, H2AC6, H2BC4, PHLDA2, CLDN7, CREB3L2, PLCXD1, LIMK2, MFSD5, SNORC, HMGN4, DDX41, FHL3, ALYREF, ZNF703, NUDT14, CRELD2, PGP, H2BC21, NRBP2, TCEAL9, UBALD2, NOTUM, TOR3A, C17orf58, TPCN1, RTN4RL2, BCAM, H1-2, AGRN, H1-0, CLDN4, S100A14, IARS1, MYL6B, NCOR2, LAMB3, FLNA, SRC, ATP6AP1-DT, ENTPD6, H2BC12, S100A6, ASPH, TXNRD1, DDX39B, MAGEA1, IPO9, GLMP, SHISA4, CCDC167, SOX18, HLA-DMA, COL5A2, PBX2, AGPAT1, FKBPL, VARS1, DDR1, HLA-F, NAT8B, LCMT1, HLA-A, MT-TL1, ARL2, SLC35F6, CLIC1, DDAH2, LYRM4, MAGEA3, CCNL2, BAIAP2-DT, RPL10P6, FTLP2, RPL10P9, PTGES3P1, APOC2, RPL39P3, HSPB1P1, RGL2, TMEM250, KMT2E-AS1, PSMB9, ARPC1A, NEAT1, H2AJ, MALAT1, TRNP1, PRKDC, LINC01419, MPV17L2, AC136475.3, SNHG9, GATD1-DT, AC006205.2, AC087482.1, DSP-AS1, PECAM1, AC009407.1, MYH4, MIR4664, AC027307.2, AL049840.5, H4C9, H2AC8, MRM1, AL606500.1

Table S2. List of downregulated unique genes for each population.

Population	Unique genes
Asian	*LAP3, PDK4, AASS, SEC62, HEBP1, TNFRSF1B, PI4K2B, TAB2, OAT, ISOC1, MAOB, LMAN1, TTC38, MCCC1, STRADB, TGDS, F11, CMTM6, COMT, SORBS1, CREM, SUN2, LGMN, DCAF11, SEC23A, LPIN2, NDFIP2, FNDC3A, SLC25A15, LONP2, FCGRT, HIBADH, ENG, PHYH, PPIF, ZNF330, KLHL2, SEPSECS, SC5D, DUSP16, SERINC1, CPEB4, EHBP1, PCYOX1, GADD45A, CTH, SDHB, MTARC2, ELL2, IRF2BPL, AVPI1, GOT1, RAMP3, ACO1, SRGN, FAM210B, TTPAL, PEPD, TMEM53, ASL, SGPP1, MCCC2, IL6ST, CTSL, NIPSNAP3A, CDK9, SLC31A1, SLCO2B1, PRCP, ITPRID2, ENPEP, HADH, SEC24B, GABARAPL1, SUOX, TMBIM6, RAB20, BNIP2, ABHD2, ALDH9A1, PIK3R1, IQGAP2, AK3, STOM, EI24, SEC24D, UBALD1, SORBS2, TSPAN7, PAFAH2, ALDH4A1, ACOX1, ZYG11B, PDLIM5, FBXO8, SFXN1, PDSS2, ARL5B, CLPX, CYB5D2, MMADHC, PPID, SUCLG2, MYD88, PCCA, SLC25A20, MAF, PER1, CSF1R, FAM167B, ACOT1, SOWAHB, CHP1, TSPYL1, ARHGEF12, ENPP1, PIM3, PAPSS2, GK, SDHD, ETFRF1, APOL6, CTSO, SRSF8, NCOA4, AL354872.2*
Caucasian	*AADAT, ABLIM3, AC008549.1, AC026803.3, AC115619.1, ACOT12, ACSM2A, ACSM3, ACSM5, AD000090.1, ADAMTS1, ADAMTSL2, ADGRG6, ADH1C, ADI1, ADRB2, AGL, AGTR1, AGXT, AHSG, AKR7A3, AL360013.3, AL391095.2, ALDOB, ALPL, AMBP, AMN, ANG, ANGPTL3, ANKS4B, ANO1, ANXA10, AOX1, APCS, APOA1, APOA5, APOC1P1, APOC3, APOH, APOL1, AR, ARSD, ART4, ASPDH, ASPG, ATF5, ATP11C, AVPR1A, AZGP1P1, BBOX1, BCHE, BGN, BHMT, C1QB, C1QTNF1, C3P1, C7, C9, CA2, CCDC3, CCDC71L, CCL19, CCL2, CCL21, CCND2P1, CDA, CDO1, CEBPD, CES3, CFHR3, CFI, CISH, CLRN3, CLU, CMBL, CNGA1, COLEC11, CPB2, CPN2, CREB3L3, CRHBP, CXCL14, CXCL2, CYB5A, CYP1A2, CYP27A1, CYP2A6, CYP2B6, CYP2C18, CYP2C19, CYP2C9, CYP2E1, CYP2J2, CYP3A4, CYP4F12, CYP4F2, CYP8B1, DCN, DCXR, DGAT2, DHRS1, DNAJC12, DPYS, DTX1, DUSP10, DUSP14, ECM1, EDNRB, EFHD1, EVA1A, F2, FABP1, FAM110C, FAM99A, FAXDC2, FCGR3A, FETUB, FGG, FGL1, FMO3, FNIP2, FOSB, FTCD, G6PC1, GADD45G, GATA4, GATM, GBP1, GBP7, GCGR, GCKR, GGT5, GJB2, GLYAT, GPAT3, GPD1, GSTA2, HAAO, HABP2, HAO2, HBA2, HES1, HGFAC, HMGCS2, HNF4A-AS1, HP, HRG, HS3ST3B1, HSD17B13, HSD17B6, IER2, IGFBP1, IGFBP2, IGHA1, IGHA2, IGHM, IL1RAP, IL27, INHBC, INMT, IRF8, ITIH1, JCHAIN, JUND, KDSR, KIAA0040, LEAP2, LECT2, LINC01018, LINC01093, LINC01702, LIPC, LRCOL1, LRG1, LY6E, MFAP4, MFSD2A, MOGAT2, MST1, MT1A, MT1DP, MT1E, MT1F, MT1G, MT1H, MT1L, MT1M, MT1X, MT-ATP8, MT-CO2, MTND2P28, MT-ND3, MT-ND6, MTTP, MYC, N4BP2L1, NAAA, NBPF13P, NCOR1, NNMT, NR0B2, NR1I2, NR4A1, NRBF2, NUGGC, OGDHL, OLFML3, ORM1, ORM2, OTC, PGLYRP2, PHGDH, PHYHD1, PIGV, PLAAT3, PLEK2, PLSCR4, PON1, PPP1R1A, PRG4, PRODH2, PROZ, PRRG4, PTMS, PTPN3, PZP, RBP4, RBP5, RETREG1, RGN, RN7SL1, RND1, RNF152, S100A8, SAA1, SAA2, SERPINA1, SERPINA10, SERPINA4, SERPINF2, SFRP5, SHBG, SIK1B, SLC10A1, SLC13A5, SLC16A2, SLC1A1, SLC22A1, SLC22A10, SLC25A47, SLC27A2, SLC27A5, SLC28A1, SLC2A2, SLC39A5, SLC4A4, SLC6A12, SLC9B2, SLCO1B1, SLCO1B3, SMIM19, SMIM24, SOCS2, SPP2, SPRY2, SRD5A2, ST3GAL6, SULT1A1, SULT2A1, TBX15, TENT5A, TF, THBS1, THRSP, TKFC, TM6SF2, TMEM45A, TMEM47, TMEM82, TMPRSS2, TTC36, TTPA, TTR, UPB1, VMO1, VNN1, XDH, ZG16*

Table S3. List of common upregulated and downregulated genes in Asian and Caucasian populations.

Expression	Genes
Upregulated	*REG3A, S100P, AKR1B10, AFP, PEG10, DUSP9, UPK3A, CPLX2, GPC3, EEF1A2, MYBL2, NQO1, MUC13, CDC20, C1QL1, BIRC5, ETV4, MMP11, UBE2C, ACSL4, LCN2, MPZ, TOP2A, ALDH3A1, SEZ6L2, PTTG1, PDZK1IP1, KRT23, SLC6A8, SPINK1, UGT2B11, CCNB1, SNCG, BAIAP2L2, SPP1, E2F1, THY1, MDK, ROBO1, CGREF1, AC092535.5, SLC51B, BEX2, SFN, PTP4A3, TRIM16L, HRCT1, CENPW, TMEM150B, C12orf75, NDUFA4L2, PLVAP, TESC, TPX2, G6PD, SNORD104, OR2I1P, SPARCL1, NOTCH3, CAP2, PDGFA, CD34, HKDC1, ZWINT, FAM83D, CDKN2C, AURKA, GMNN*
Downregulated	*FCN2, MARCO, FCN3, HAMP, OIT3, CD5L, NAT2, MT2P1, DNASE1L3, IGFALS, APOF, ADH4, GHR, CYP2C8, RND3, CYP39A1, CXCL12, RCAN1, UROC1, PHLDA1, PCK1, LCAT, GBA3, KDM8, FOS, MT2A, LPA, EGR1, CSRNP1, CYP4A11, CYP4A22, TCIM, RDH16*

Table S4. The top 20 enriched biological processes associated with common upregulated genes in both populations.

Category	Biological Processes Unique
Biological Process	• mRNA splicing, via spliceosome (GO: 0000398)<br>• regulation of DNA-templated DNA replication initiation (GO: 0030174)<br>• DNA unwinding involved in DNA replication (GO: 0006268)<br>• double-strand break repair via break-induced replication (GO: 0000727)<br>• U2-type prespliceosome assembly (GO: 1903241)<br>• spliceosomal snRNP assembly (GO: 0000387)<br>• cell division (GO: 0051301)<br>• 7-methylguanosine cap hypermethylation (GO: 0036261)<br>• regulation of cell cycle (GO: 0051726)<br>• chaperone-mediated gene folding (GO: 0061077)<br>• positive regulation of mitotic metaphase/anaphase transition (GO: 0045842)<br>• RNA splicing (GO: 0008380)<br>• DNA replication initiation (GO: 0006270)<br>• heterochromatin formation (GO: 0031507)<br>• mRNA processing (GO: 0006397)<br>• positive regulation of DNA-templated transcription (GO: 0045893)<br>• nuclear pore localization (GO: 0051664)<br>• nuclear envelope organization (GO: 0006998)<br>• anaphase-promoting complex-dependent catabolic process (GO: 0031145)<br>• regulation of mitotic cell cycle (GO: 0007346)
Cellular Component	• nucleoplasm (GO: 0005654)<br>• cytosol (GO: 0005829)<br>• nucleus (GO: 0005634)<br>• spliceosomal complex (GO: 0005681)<br>• cytoplasm (GO: 0005737)<br>• U2-type precatalytic spliceosome (GO: 0071005)<br>• extracellular exosome (GO: 0070062)<br>• U4/U6 x U5 tri-snRNP complex (GO: 0046540)<br>• precatalytic spliceosome (GO: 0071011)<br>• U1 snRNP (GO: 0005685)<br>• CMG complex (GO: 0071162)<br>• MCM complex (GO: 0042555)<br>• nuclear laminanuclear lamina (GO: 0005652)<br>• chromosome, telomeric region chromosome, telomeric region (GO: 0000781)<br>• spindle (GO: 0005819)<br>• small nuclear ribonucleogene complex (GO: 0030532)<br>• U2 snRNP (GO: 0005686)<br>• U2-type spliceosomal complex (GO: 0005684)<br>• gene folding chaperone complex (GO: 0101031)<br>• catalytic step 2 spliceosome (GO: 0071013)
Molecular Function	• gene binding (GO: 0005515)<br>• RNA binding (GO: 0003723)<br>• identical gene binding (GO: 0042802)<br>• gene homodimerization activity (GO: 0042803)<br>• gene kinase binding (GO: 0019901)<br>• ubiquitin binding (GO: 0043130)

	• gene heterodimerization activity (GO: 0046982)<br>• cadherin binding (GO: 0045296)<br>• ATP-dependent gene binding (GO: 0043008)<br>• single-stranded DNA helicase activity (GO: 0017116)<br>• gene-containing complex binding (GO: 0044877)<br>• DNA polymerase binding (GO: 0070182)<br>• ATP-dependent H2AZ histone chaperone activity (GO: 0140849)<br>• DNA binding (GO: 0003677)<br>• tubulin binding (GO: 0015631)<br>• U1 snRNP binding (GO: 1990446)<br>• U1 snRNA binding (GO: 0030619)<br>• structural constituent of ribosome (GO: 0003735)<br>• histone deacetylase binding (GO: 0042826)<br>• unfolded gene binding (GO: 0051082)

Table S5. The top 20 enriched biological processes associated with common down-regulated genes in both populations.

Category	Biological Processes Unique
Biological Process	• fibrinolysis (GO: 0042730)<br>• complement activation, classical pathway (GO: 0006958)<br>• fatty acid beta-oxidation (GO: 0006635)<br>• fatty acid beta-oxidation using acyl-CoA dehydrogenase (GO: 0033539)<br>• zymogen activation (GO: 0031638)<br>• response to hypoxia (GO: 0001666)<br>• lipid metabolic process (GO: 0006629)<br>• electron transport coupled proton transport (GO: 0015990)<br>• gluconeogenesis (GO: 0006094)<br>• response to organic cyclic compound (GO: 0014070)<br>• complement activation, lectin pathway (GO: 0001867)<br>• cellular response to insulin stimulus (GO: 0032869)<br>• response to starvation (GO: 0042594)<br>• aerobic respiration (GO: 0009060)<br>• retinoic acid metabolic process (GO: 0042573)<br>• cell surface pattern recognition receptor signaling pathway (GO: 0002752)<br>• fatty acid metabolic process (GO: 0006631)<br>• bile acid biosynthetic process (GO: 0006699)<br>• response to xenobiotic stimulus (GO: 0009410)<br>• regulation of keratinocyte differentiation (GO: 0045616)
Cellular Component	• mitochondrion (GO: 0005739)<br>• mitochondrial matrix (GO: 0005759)<br>• blood microparticle (GO: 0072562)<br>• extracellular exosome (GO: 0070062)<br>• peroxisomal matrix (GO: 0005782)<br>• extracellular space (GO: 0005615)<br>• peroxisome (GO: 0005777)<br>• cytosol (GO: 0005829)<br>• extracellular region (GO: 0005576)<br>• platelet alpha granule lumen (GO: 0031093)<br>• collagen-containing extracellular matrix (GO: 0062023)<br>• mitochondrial membrane (GO: 0031966)<br>• external side of plasma membrane (GO: 0009897)<br>• transcription factor AP-1 complex (GO: 0035976)<br>• respiratory chain complex I (GO: 0045271)<br>• membrane attack complex (GO: 0005579)<br>• serine-type endopeptidase complex (GO: 1905370)<br>• mitochondrial inner membrane (GO: 0005743)<br>• RNA polymerase II transcription regulator complex (GO: 0090575)<br>• endoplasmic reticulum membrane (GO: 0005789)
Molecular Function	• oxidoreductase activity (GO: 0016491)<br>• heme binding (GO: 0020037)<br>• acyl-CoA dehydrogenase activity (GO: 0003995)<br>• pyridoxal phosphate binding (GO: 0030170)<br>• identical gene binding (GO: 0042802)<br>• flavin adenine dinucleotide binding (GO: 0050660)

	• oxidoreductase activity, acting on the CH-CH group of donors (GO: 0016627)<br>• acetyl-CoA C-acyltransferase activity (GO: 0003988)<br>• all-trans-retinol dehydrogenase (NAD+) activity (GO: 0004745)<br>• electron transfer activity (GO: 0009055)<br>• alcohol dehydrogenase (NAD+) activity (GO: 0004022)<br>• NADH dehydrogenase (ubiquinone) activity (GO: 0008137)<br>• NAD binding (GO: 0051287)<br>• signaling receptor binding (GO: 0005102)<br>• NADPH binding (GO: 0070402)<br>• acetyl-CoA C-acetyltransferase activity (GO: 0003985)<br>• serine-type endopeptidase activity (GO: 0004252)<br>• gene homodimerization activity (GO: 0042803)<br>• long-chain fatty acid omega-hydroxylase activity (GO: 0102033)<br>• oxidoreductase activity, acting on paired donors, with incorporation or reduction of molecular oxygen (GO: 0016705)
