# Supplementary material for: Racial disparities in hepatocellular carcinoma: a TCGA-based gene expression study of Caucasian and Asian populations
Source: Explor Target Antitumor Ther. 2025 Nov 2;6:1002344. doi: 10.37349/etat.2025.1002344 (PMC12597399; doi:10.37349/etat.2025.1002344)
Supplement: Supplementary file 2 [file 1002344_sup_2.pdf]

Supplementary Figures

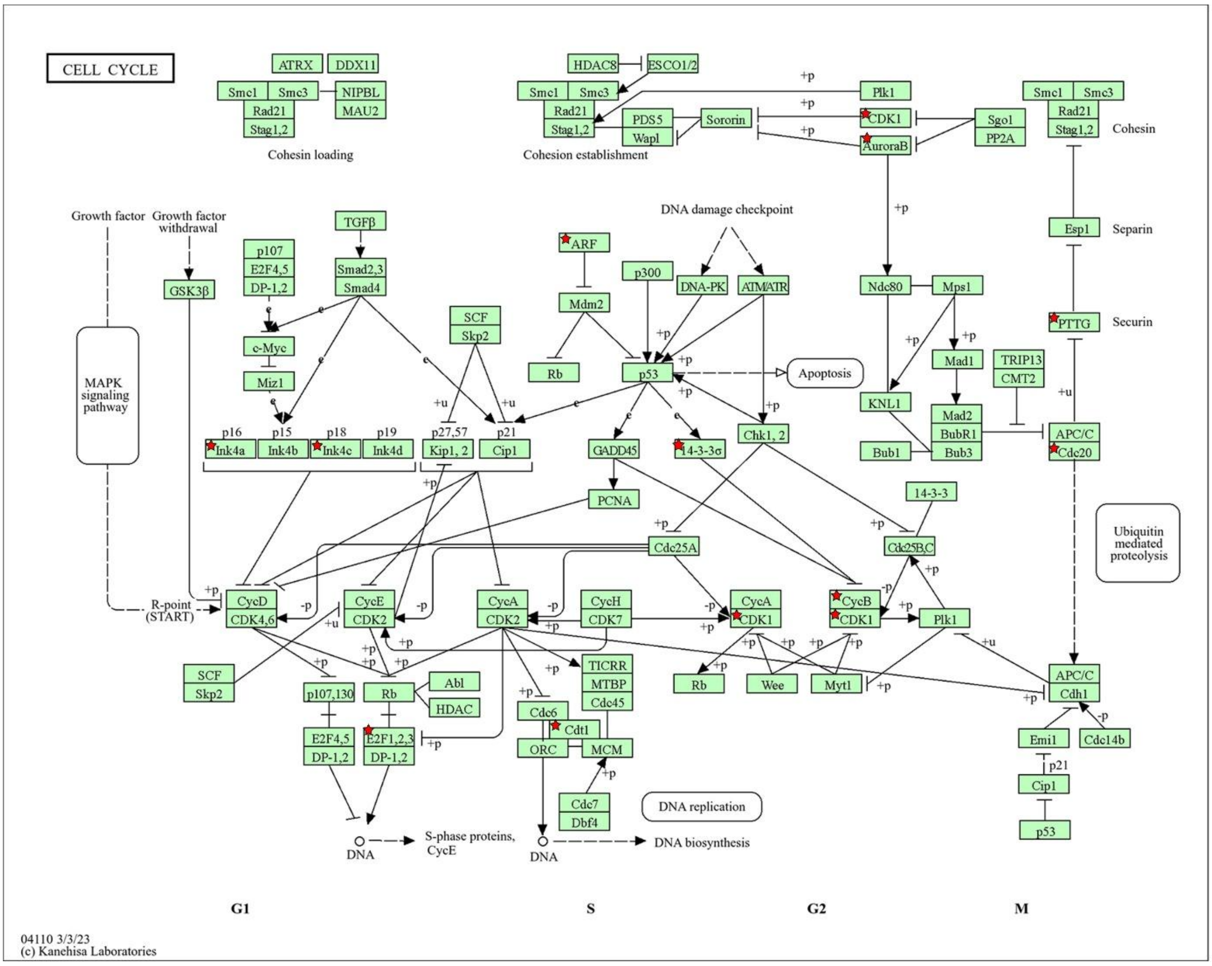

Figure S1. KEGG cell cycle pathway enriched in Asian tumors compared to controls (FDR = 7.2 × 10^{-5}). Genes marked with red stars represent significantly upregulated genes in tumor samples. Upregulation is mainly observed at the G1/S transition and DNA damage checkpoint (CDK2, Cdc6, Chk1/Chk2, GADD45, and Cdc20), indicating accelerated S-phase entry, checkpoint adaptation, and enhanced proliferative signaling in Asian tumors.

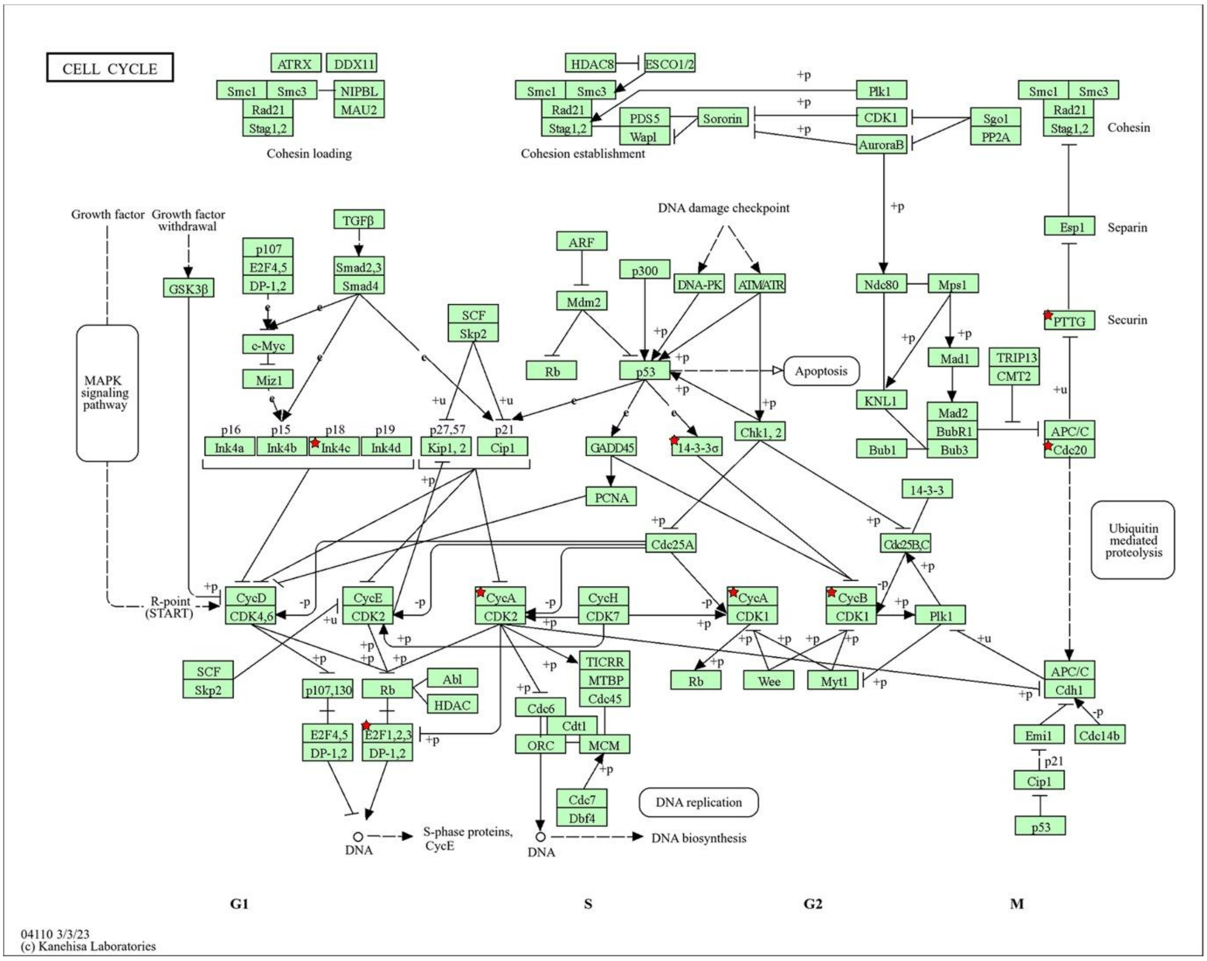

Figure S2. KEGG cell cycle pathway enriched in Caucasian tumors compared to controls (FDR = 0.076). Genes marked with red stars represent significantly upregulated genes in tumor samples. Increased expression is observed in cyclin-CDK complexes, mitotic regulators (CDK1, Plk1, Cdc20), and the APC/C ubiquitin-mediated proteolysis system, reflecting enhanced mitotic regulation and balanced cell cycle progression in Caucasian tumors.

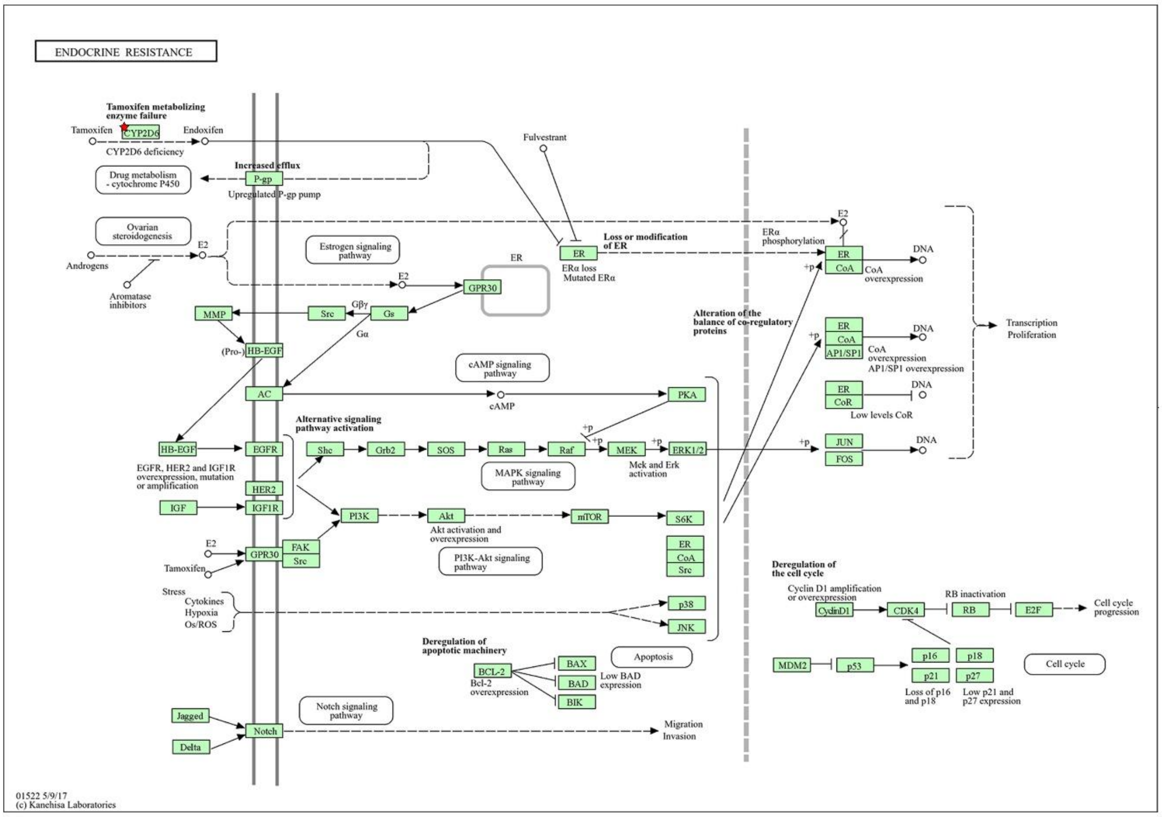

Figure S3. KEGG endocrine resistance pathway enriched in Asian tumors compared to Caucasians (FDR = 0.15). Genes marked with red stars represent significantly upregulated genes in Asian tumors. Upregulation of CYP2D6 and downstream activation of the PI3K-Akt and MAPK signaling cascades indicate enhanced alternative survival signaling and potential resistance to endocrine (hormone) therapy.

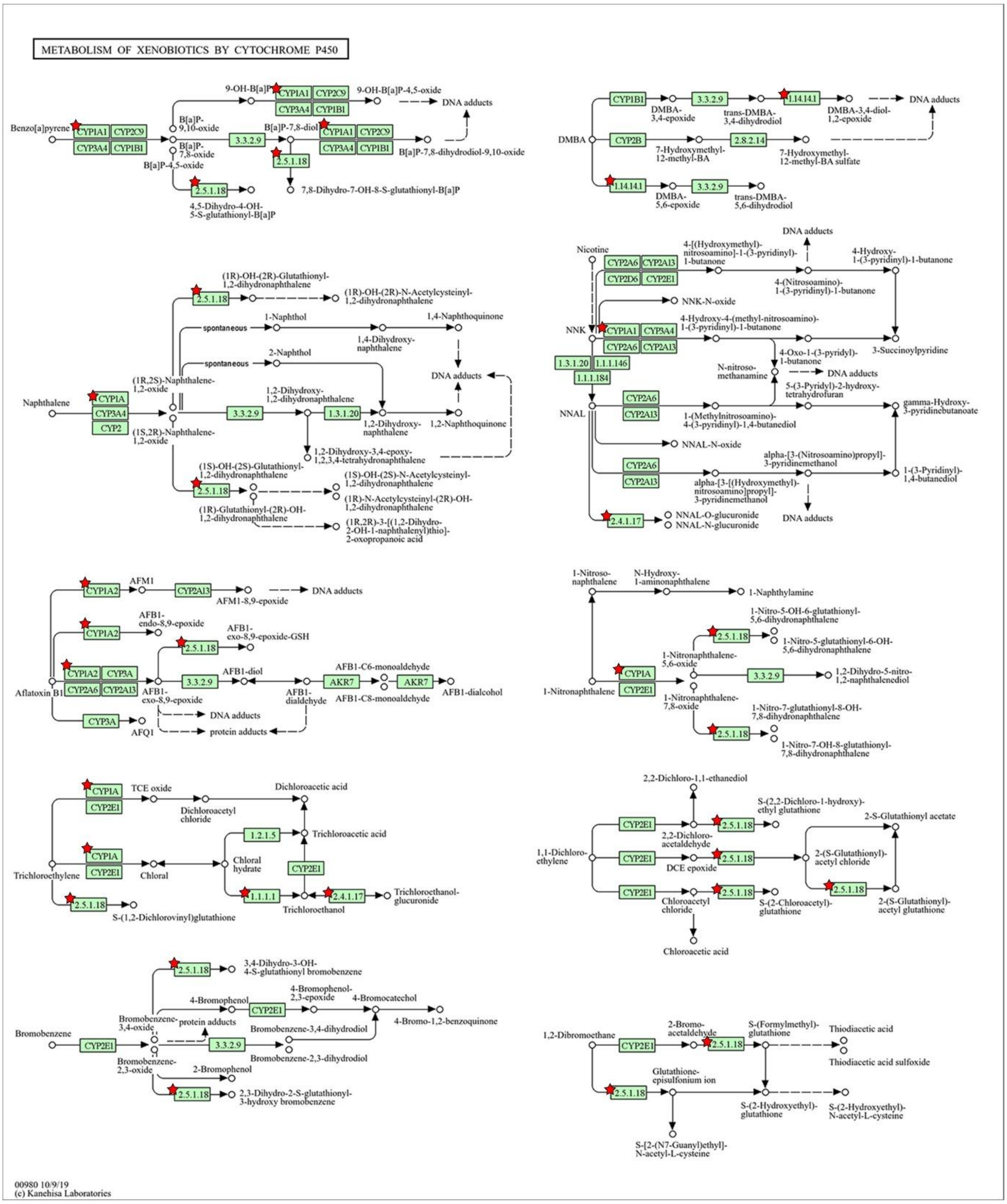

Figure S4. KEGG metabolism of xenobiotics by cytochrome P450 pathway enriched in Asian tumors compared to controls (FDR = 2.0 × 10^{-6}). Genes marked with red stars represent significantly upregulated genes in Asian tumors. Prominent induction of cytochrome P450 enzymes (CYP1A1, CYP1A2, CYP2E1, CYP3A4) and glutathione S-transferases (GSTA1, GSTP1) suggests enhanced xenobiotic metabolism and detoxification capacity in Asian tumor samples.
